# IL-20R Activation via rIL-19 Enhances Hematoma Resolution through the IL-20R1/ERK/Nrf2 Pathway in an Experimental GMH Rat Pup Model

**DOI:** 10.1155/2021/5913424

**Published:** 2021-01-19

**Authors:** Shengpeng Liu, Jerry J. Flores, Bo Li, Shuixiang Deng, Gang Zuo, Jun Peng, Jiping Tang, John H. Zhang

**Affiliations:** ^1^Department of Pediatrics, Shenzhen People's Hospital, the Second Clinical Medical College of Jinan University, Shenzhen, Guangdong, China; ^2^Department of Physiology and Pharmacology, Loma Linda University School of Medicine, Loma Linda, California, USA; ^3^Department of Critical Care Medicine, Huashan Hospital, Fudan University, 12 Middle WuLuMuQi, Shanghai, China; ^4^Departments of Anesthesiology, Neurosurgery and Neurology, Loma Linda University School of Medicine, Loma Linda, California, USA

## Abstract

**Aims:**

Blood clots play the primary role in neurological deficits after germinal matrix hemorrhage (GMH). Previous studies have shown a beneficial effect in blood clot clearance after hemorrhagic stroke. The purpose of this study is to investigate interleukin-19's role in hematoma clearance after GMH and its underlying mechanism of IL-20R1/ERK/Nrf2 signaling pathway.

**Methods:**

A total of 240 Sprague-Dawley P7 rat pups were used. GMH was induced by intraparenchymal injection of bacterial collagenase. rIL-19 was administered intranasally 1 hour post-GMH. IL-20R1 CRISPR was administered intracerebroventricularly, or Nrf2 antagonist ML385 was administered intraperitoneally 48 hours and 1 hour before GMH induction, respectively. Neurobehavior, Western blot, immunohistochemistry, histology, and hemoglobin assay were used to evaluate treatment regiments in the short- and long-term.

**Results:**

Endogenous IL-19, IL-20R1, IL-20R2, and scavenger receptor CD163 were increased after GMH. rIL-19 treatment improved neurological deficits, reduced hematoma volume and hemoglobin content, reduced ventriculomegaly, and attenuated cortical thickness loss. Additionally, treatment increased ERK, Nrf2, and CD163 expression, whereas IL-20R1 CRISPR-knockdown plasmid and ML385 inhibited the effects of rIL-19 on CD163 expression.

**Conclusion:**

rIL-19 treatment improved hematoma clearance and attenuated neurological deficits induced by GMH, which was mediated through the upregulation of the IL-20R1/ERK/Nrf2 pathways. rIL-19 treatment may provide a promising therapeutic strategy for the GMH patient population.

## 1. Introduction

Germinal matrix hemorrhage (GMH) is defined by the rupture of thin-walled blood vessels located in the germinal matrix of preterm/low-birthweight infants [1, 2]. It is one of the leading causes of morbidity and mortality among preterm infants [3]. Survivors suffer from severe long-term neurological, motorial, and cognitive deficits, which contribute to cerebral palsy, developmental delays, mental retardation, and posthemorrhagic hydrocephalus (PHH) [4, 5]. Currently, there are no safe-effective drug therapies to treat the GMH patient population. Thus, a safe and noninvasive strategy is essential in the management for GMH.

After the rupture of blood vessels, blood components permeate into the brain parenchyma [6, 7]. Cytotoxins such as heme, peroxiredoxin 2 (PRX-2), and iron are released from metabolized blood cell (RBC) causing secondary brain injury through the increase in neuroinflammation, oxidative stress, neuronal death, and/or apoptosis [8, 9]. Additionally, the intracerebroventricular blood clot directly impairs the circulation and absorption of the cerebrospinal fluid (CSF), leading to the development of PHH [10].

Microglia play a pivotal role in the pathological process after injury, through the regulation of the immune response, oxidative stress, and the removal of blood products or tissue debris [11]. Within the neonatal CNS, microglia play a positive role in blood-brain development and postnatal development and were shown to be neuroprotective after germinal matrix hemorrhage [2]. In a neonatal model of GMH, microglia/macrophages participate in the phagocytosis of red blood cells (RBCs) which enhanced hematoma resolution. The quick resolution of hematoma resulted in the reduction of ventricular dilation and improved long-term neurobehavior [12].

Scavenger receptor CD163, a class B glycoprotein, has been shown to induce microglia/macrophage phagocytosis which induced hematoma clearance [11, 13]. CD163 is primarily expressed on alternatively activated microglia/macrophages (M2) [14]. The CD163 function in the neonatal CNS is yet to be evaluated.

Interleukin-19 (IL-19), part of the IL-10 subfamily which includes IL-10, IL-20, IL-22, and IL-24, has been shown to be neuroprotective after stroke [15–17]. IL-19 binds to interleukin-20 receptor (IL-20R) type 1, which is composed of two subunits (IL-20R1 and IL-20R2 or IL-20R*α* and IL-20R*β*), whereas other ligands such as IL-20 and IL-24 bind to both type 1 and type 2 receptors (IL-22R1 and IL-20R2) [18]. Extracellular signal-regulated kinase 1/2 (ERK1/2), a prototypic subfamily of MAPKs, has been recognized as a downstream target after IL-20R activation [18, 19]. Current literatures suggest that ERK1/2 may increase the expression of nuclear factor erythroid 2-related factor 2 (Nrf2), which has been shown to enhance hematoma resolution by increasing CD163 in microglia/macrophages [20, 21].

Because of the aforementioned evidence, we hypothesized that recombinant IL-19 treatment increases hematoma resolution, expression of scavenger receptors on microglia/macrophages, M2 microglia, reduces ventricular dilatation, and improves short- and long-term neurological deficits and that the beneficial effects may be mediated by the activation of the IL-20R1/ERK/Nrf2/CD163 signaling pathway.

## 2. Materials and Methods

### 2.1. Animals and GMH Model

All experimental procedures were approved by the Institutional Animal Care and Use Committee at Loma Linda University, and in accordance with the National Institute of Health guidelines for the treatment of animals. A total of two hundred and forty seven-day-old (P7) female and male Sprague-Dawley (SD) neonatal rats were purchased from Envigo (Livermore, CA, US). P7 pups were used for the GMH model as their brains are comparable to 30-32 weeks of human gestation. We experienced zero mortality of rat pups during these experiments. Upon arrival or after surgeries, pups remained with the mother until euthanasia or the weaning process for long-term experiments. After being weaned, two pups of the same gender were housed per cage. All animals are housed on a 12 : 12 hour light/dark cycle in controlled appropriate humidity and temperatures with plenty of food and water.

The GMH model was induced by stereotactic-guided injection of bacterial collagenase as previously described in [22]. Neonatal rat pups were anesthetized with 5% isoflurane and placed onto a stereotactic head frame with a neonatal adaptor in a prone position. A scalp incision is made along the midline exposing the bregma. A burr hole is drilled on the right side of the skull at coordinates of 1.6 mm (right lateral), 1.5 mm (rostral), and dura of 2.7 mm (depth) from bregma. 0.3 units of collagenase VII-S (VII-S, Sigma; 0.3 U in 0.5 *μ*l of PBS) was infused by a 10 *μ*l syringe (Hamilton Co. US) guided by a microinfusion pump (Harvard Apparatus, Hollison, MA). To minimize the leakage of collagenase from the burr hole, the syringe was kept in place for 5 minutes after injection and was withdrawn at a speed of 0.5 mm/min.

Bone wax was used to seal the burr hole, and a suture was used to close the incision line. Lastly, the pups were placed on a 37°C heated blanket for recovery, and after recovery, the pups were placed back with the dam. During the operation, the skin color, temperature, respiratory rhythm and frequency, and heart rates were closely monitored. The procedure for sham groups were similar to the vehicle groups, yet they were only subjected to needle insertion without collagenase injection.

### 2.2. Experimental Design and Groups

**Figure 1:**
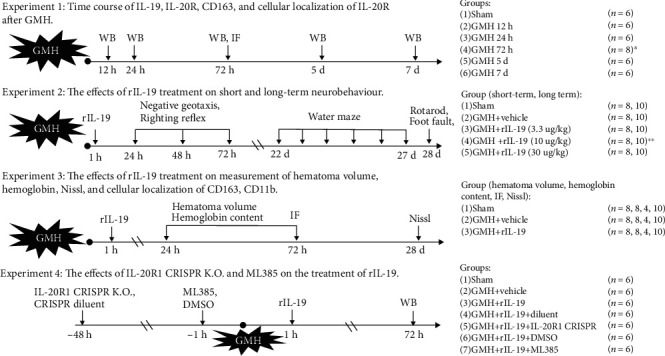
Experimental design and animal groups. ∗Extra 2 pups were used for IF (IL-20R1, IL-20R2, and CD11b) at 72 hours after GMH. ∗∗Assumed the optimal dose group.

Both male and female pups were randomized into the following experimental groups: sham, GMH + vehicle, GMH + rIL19, GMH + rIL-19 + IL-20R1 CRISPR (knockdown), GMH + rIL-19 + IL-20R1 CRISPR diluent, MH + rIL-19 + ML385, and MH + rIL-19 + DMSO.  Figure 1 shows each animal group and experimental design.

**Experiment 1 .**

Experiment 1 was performed to detect the endogenous levels of IL-19, IL-20R1, IL-20R2, and CD163. Western blot (WB) was used to analyze the expression of these aforementioned proteins in the whole brain at 12 h, 24 h, 72 h, 5 d, and 7 d after GMH. To confirm if the complex of IL-20 receptor type 1 (IL-20R1 and IL-20R2) is expressed on microglia, the colocalization of IL-20R (IL-20R1, IL-20R2) and CD11b was conducted for immunofluorescence.

**Experiment 2 .**

Experiment 2 was performed to investigate the effects of rIL-19 treatment on short- and long-term neurobehavior after GMH. Negative geotaxis and righting reflex were used to test short-term neurobehavior deficits. The Morris water maze, rotarod, and foot fault were used to assess long-term neurobehavior deficits.

**Experiment 3 .**

Experiment 3 was performed to observe the effects of rIL-19 on hematoma resolution after GMH. Hematoma volume, hemoglobin content, ventricular volume, and relative cortical thickness were evaluated.

**Experiment 4 .**

Experiment 4 was performed to investigate the mechanism of rIL-19 treatment on IL-20R1 signaling pathway. IL-20R1 inhibitor, IL-20R1 CRISPR (K.O.), and Nrf2 inhibitor ML385 were used. Expression levels of IL-20R1, p-ERK, Nrf2, and CD163 were detected by WB.

### 2.3. Drug Administration

rIL-19 (4546, Biovision, USA) at dosages of 3.3, 10, and 30 *μ*g/kg (best dose response) or dH20 (shame and vehicle) were intranasally administered at 1 h postictus and given for three consecutive days. The dosage concentrations for rIL-19 were adapted from the following experimental articles [23, 24]. IL-20R1 CRISPR knockout (sc-437367, Santa Cruz, USA) plasmids (r) or CRISPR diluent at dosage of 1 *μ*g/pup were intracerebroventricularly administered 48 h prior to GMH surgery. Nrf2 inhibitor ML385 (30 mg/kg) [25] or 5% dimethyl sulfoxide (DMSO) was intraperitoneally administered 1 h before surgery, and every 24 h for 3 consecutive days.

### 2.4. Neurobehavioral Examination

Negative geotaxis and righting reflex were employed to examine the short-term neurobehavior and water maze, foot fault, and rotarod test for long-term neurobehavior as previously reported in [22]. All of tests were executed in a blinded manner.

#### 2.4.1. Negative Geotaxis

Neonatal pups were placed on a 45° rough ramp in a downward orientation, and the time it takes for the pup to recognize that it is on an incline and to correct its positioning by making a 180-degree turn facing upwards within a 60-second duration is recorded. This test was repeated three times for three consecutive days post-GMH, and the average of each day was taken for statistical analysis.

#### 2.4.2. Righting Reflex

The pups were placed supinely on a horizontal plate, and the time it takes for the pup to flip/rollover was recorded within a 20-second duration. Every pup also was examined in repeats of three as described for negative geotaxis.

#### 2.4.3. Morris Water Maze

The Morris water maze was performed to evaluate spatial and reference memory at 22 and 27 days postictus as previously described in [26, 27]. After the training block trial, rats undergo 4 trial blocks to find the platform. The time and distance it takes the animal to find the platform was recorded. On the last day, the platform is removed to perform the probe trial, where the time spent swimming in the quadrant where the platform is located is measured.

#### 2.4.4. Rotarod and Foot Fault Test

Rotarod and foot fault tests were conducted to evaluate the motor, locomotor, and balance after injury and treatment as previously described in [28, 29]. Rats were placed on an accelerating rod, which is 70 mm in diameter, at starting speeds of 5 RPM or 10 RPM. Falling latency was recorded by the Rotor Rod instrument (SD Instruments, San Diego, CA). During the foot fault test, animals were placed on a grid wire (0.6 × 1.5 m, 2.5 cm apart) where the number of missteps were recorded in a one-minute interval.

### 2.5. Hematoma Volume and Hemoglobin Content Measurement

Hematoma measurement was adapted from the following manuscript [12]. Rats were anesthetized by 5% isoflurane, followed by transcardiac perfusion with 4°C phosphate-buffered saline (PBS). The whole brain was extracted and cut into 5 coronal sections equidistantly. The ImageJ software (NIH) was used to count the area of hematoma (mm^2^), and then the hematoma volume was estimated with the following formula: hematoma volume (mm^3^) = area of every section (mm^2^) × section number × thickness (mm).

Hemoglobin assay was conducted to measure hemoglobin content as previously reported in [30]. Extracted forebrain tissue was placed into glass tubes with 3 mL of PBS. The brains were homogenized for one minute (Fisher Scientific, USA) followed by ultrasonication for one minute to lyse erythrocyte membranes. After centrifugation for thirty minutes (2°C), the supernatant was extracted from the sample. Drabkin's reagent (Sigma-Aldrich) is combined with supernatant at a 4 : 1 ratio and left to react for 15 minutes at room temperature. Using a spectrophotometer (540 nm; Genesis 10UV; Thermo Fisher Scientific), absorbance was calculated into hematoma volume (*μ*L) as described in [12].

### 2.6. Ventricular Volume and Cortical Thickness Estimation

At 28 days postictus, animals were euthanized, and the whole brains were extracted and prepared for histology. To be better observed and measured, histological slices were stained by Nissl. Ventricular volume and cortical thickness were evaluated as previously described in [31]. The brains were embedded with Optimal Cutting Temperature (OCT, Fisher Scientific, Waltham, MA) and sectioned into 20 *μ*m thick slices (Leica Microsystems, LM3050S). Samples were stained with Cresyl violet, and optical dissector principles were used to delineate cerebral structure borders as described in [32]. Ventricular volume and cortical thickness were analyzed using the ImageJ software (NIH) as previously described in [33].

### 2.7. Western Blot

The rat forebrains were extracted and the target proteins were detected by Western blot as previously described in [34]. Briefly, both hemispheres were homogenized in RIPA lysis buffer (sc-24948, Santa Cruz, USA) and then centrifuged at 14,000 rpm for 30 minutes. Protein samples (4 *μ*L, 1.5 mg/mL) were loaded on 7.5-15% tris-glycine gel, separated by SDS-PAGE, and transferred to 0.2 or 0.45 *μ*m nitrocellulose membrane. Membranes were incubated overnight at 4°C with the following primary antibodies: mouse monoclonal anti-IL-19 (1 : 1000, LS-C663662, LSBio); rabbit polyclonal anti-IL20R2 (1 : 1000, bs-2620R, Bioss); rabbit polyclonal anti-IL20R1 (1 : 1000, bs-2619R, Bioss); rabbit monoclonal anti-p-ERK (1 : 1000, Santa Cruz); mouse monoclonal anti-ERK (1 : 2000, sc-514302, Santa Cruz); rabbit polyclonal anti-Nrf2 (1 : 1000, GTX103322, Gene Tex); and mouse monoclonal anti-CD163 (1 : 1000, GTX42366, Gene Tex). The same membranes were probed with an antibody against *β*-actin (1 : 5000, ab8226, Abcam) as an internal control. After washing the membranes, the following corresponding secondary antibodies were used to incubate the membranes for 2 h at room temperature: anti-mouse (1 : 5000, SC-516102, Santa Cruz); anti-rabbit (1 : 3000, Santa Cruz). The membrane was then exposed to radiography films to display the protein bands. Finally, the density of bands was analyzed for the relative density of the resultant protein immunoblot by the ImageJ software (NIH).

### 2.8. Immunofluorescence

Immunofluorescence staining was performed as previously described in [35]. The animals were sacrificed and transcardially perfused with PBS followed by 4% formalin. They were then stored in 4% formalin at 4°C for a week and transferred to 30% sucrose solution until all samples were dehydrated. Samples were then prepared in OCT and sectioned to 20 *μ*m thick slices. Slices were stained with the following primary antibodies: IL20R1 (1 : 100, Bioss), IL20R2 (1 : 100, Bioss), CD163 (1 : 100,), and CD11b (1 : 200, Abcam) at 4°C overnight. They were then incubated with the appropriate fluorescence-conjugated secondary antibodies (1 : 200, Jackson ImmunoResearch Labs) for 1 hour at room temperature followed by dropping DAPI. The perihemorrhagic area was imaged by a DMi8 fluorescent microscope (Leica Microsystems, Baffalo Grove, IL) under a 400× fold field. To detect the proportion of CD163 positive cells in microglia/macrophages, 4 fields from the surrounding area of perihematomal region were taken into been counted.

### 2.9. Rigor and Statistical Analysis

All animals in this study were numbered and randomly sorted into each of the experimental animal groups using Excel. The protocol of randomization by Excel is as follows: (1) sequence the pups vertically in an Excel spreadsheet; (2) use “RAND” function to generate a random number for each pup; (3) rerank the random numbers in an ascending or descending order; and (4) designate a certain number of pups into each experimental group according to the study design. All experimental groups and identifying numbers were unknown to the surgeons and researchers conducting methodology.

Animal sample size evaluation was determined using a type I error rate of 0.05 and a power of 0.8 on a 2-sided test by power analysis. Parametric data was described as mean ± SEM and analyzed using one-way ANOVA followed by Tukey's post hoc test. Longitudinal data were analyzed using two-way repeated measure ANOVA with Tukey's post hoc test. The counting data was tested using chi-square test. *P* values of < 0.05 were considered statistically significant. GraphPad Prism 7 (La Jolla) were used for graphing and analyzing all the data.

## 3. Results

### 3.1. Endogenous Expression of IL-19, IL-20R1, IL-20R2, and CD163 Increased after GMH

**Figure 2:**
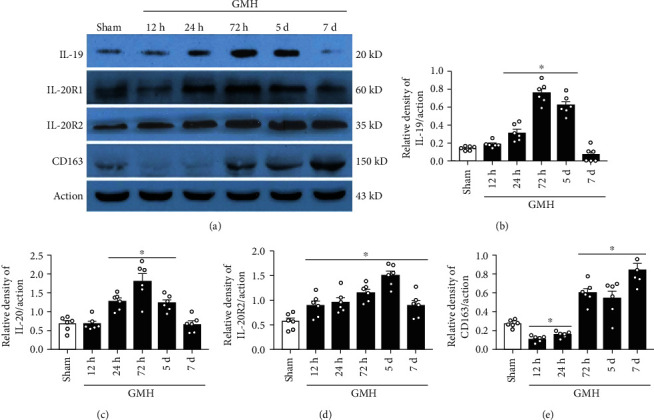
The time-course of endogenous levels of IL-19, IL-20R1, IL-20R2, and CD163 after GMH. (a) Representative bands of IL-19, IL-20R1, IL-20R2, CD163, and Actin. (b) The expression of IL-19 at various time-points. (c) The expression of IL-20R1 at different time-points after GMH. (d) The expression of IL-20R2 at different time-points after GMH. (e) The expression of CD163 at different time-points after GMH. ∗*P* < 0.05 vs. sham, mean ± SEM, one-way ANOVA, Tukey's test, *n* = 6/group.

The endogenous proteins were examined by WB at 0 (sham), 12, 24, and 72 hours, and 5 and 7 days post-GMH. Endogenous IL-19 expression was significantly increased at 24 hours to 5 days, where peak expression was seen at 72 hours after GMH (Figures 2(a) and 2(b)). Both subunits of IL-20R expression increased after GMH, where IL-20R1 increased at 24 hours to 5 days, and peak expression was achieved at 72-hour time point (Figures 2(a) and 2(c)), while IL-20R2 increased at 12 hours to 7 days, and peak expression was seen at 5 days after GMH (Figures 2(a) and 2(d)). The endogenous expression of CD163 decreased at 12 and 24 hours and then increased significantly at 72 hours to 7 days postictus (Figures 2(a) and 2(e)). Based on these results, the 3-day time point was chosen to evaluate the mechanism of action.

**Figure 3:**
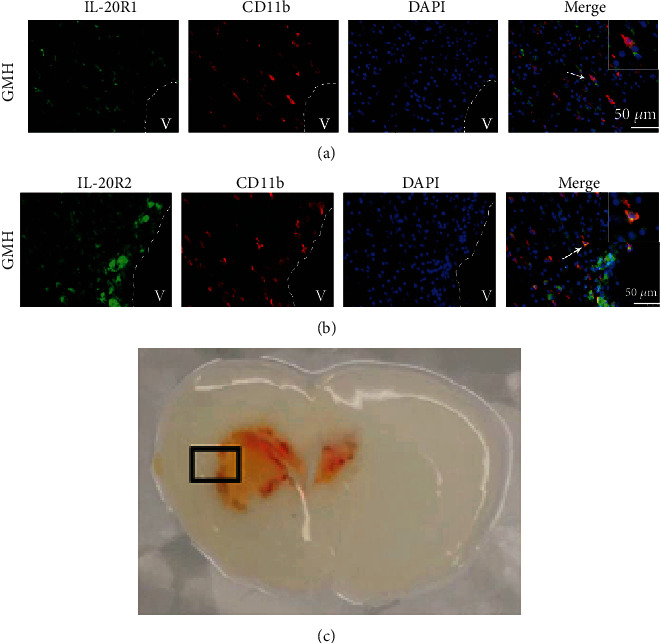
Colocalization of IL-20R1 or IL-20R2 with CD11b at 72 hours after GMH. (a) Colocalization of IL-20R1 with CD11b. (b) Colocalization of IL-20R2 with CD11b. (c) Representative GMH brain indicates where IHC images were taken. Scale bar = 50 *μ*m, *n* = 2/group.

Double immunofluorescence staining was used to determine the cellular localization of IL-20R1 and IL-20R2 in the CNS at 72 hours after GMH. Both IL-20R1 and IL-20R2 were found to be colocalized with CD11b at the site of perihematoma (Figures 3(a) and 3(c)). These results indicate that IL-20R type 1 is expressed on microglia/macrophage cells.

### 3.2. Intranasal Administration of Murine Recombinant IL-19 Improved Short- and Long-Term Behavioral Outcomes after GMH

**Figure 4:**
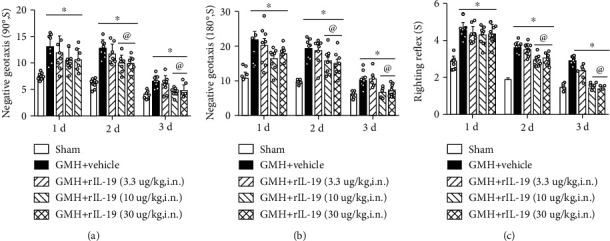
Intranasal administration of rIL-19 improved short-term neurological function at 48 and 72 hours after GMH. (a) 90° negative geotaxis; (b) 180° negative geotaxis; (c) righting reflex. ∗*P* < 0.05 vs. sham, ^@^*P* < 0.05 vs. GMH + vehicle, mean ± SEM, two-way repeated measurement ANOVA, Tukey's test, *n* = 8/group.

Three different dosages of rIL-19 (3.3 *μ*g/kg, 10 *μ*g/kg, and 30 *μ*g/kg) were administered intranasally to determine the best dose response. Animals were randomly divided into the following groups: Sham, GMH + vehicle, GMH + low dosage rIL-19 (3.3ug/kg), GMH + middle dosage rIL-19 (10ug/kg), and GMH + high dosage rIL-19 (30ug/kg) animal group. When compared to sham, all GMH groups had significantly short-term neurological impairments. At the 24-hour time point after GMH, middle and high dosage of rIL-19 significantly improved motor coordination (negative geotaxis) when compared to the vehicle group (Figures 4(a) and 4(b)), yet righting reflex demonstrated no significant difference. At 48 and 72 hours after GMH, middle and high dosage groups significantly improved short-term behavior when compared to the vehicle group (Figures 4(a)–4(c)). Both middle and high dosages of rIL-19 improved short-term neurological deficits, and the medium dosage (10 *μ*g/kg) was selected as the best dose.

**Figure 5:**
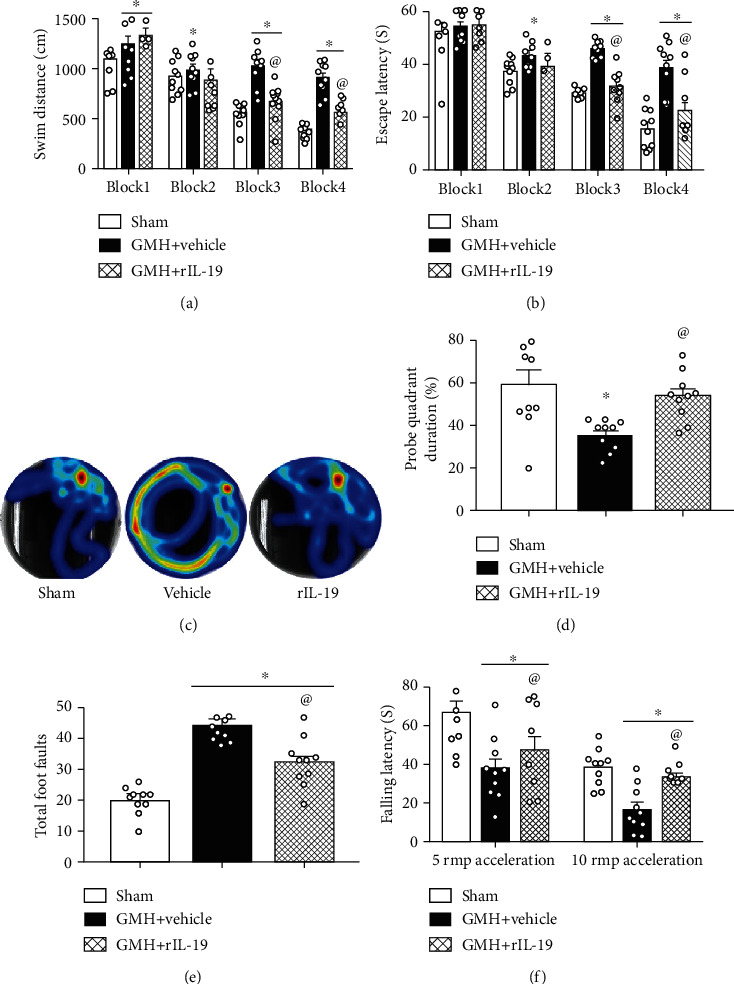
rIL-19 (10 *μ*g/kg, i.n.) treatment improved memory and motor function at 22-27 days after GMH. (a–d) Water maze tests, (e) foot fault tests, and (f) rotarod tests. ∗*P* < 0.05 vs. sham, ^@^*P* < 0.05 vs. vehicle, mean ± SEM, two-way repeated measurement ANOVA, one-way ANOVA for (d–f), Tukey's test, *n* = 10/group.

To assess the effects of rIL-19 on long-term neurological function (spatial memory, reference memory, and motor coordination), neurobehavioral tests were performed at 22 to 27 days after GMH. No significant difference was seen in escape latency and swim distance among all animal groups at blocks 1 and 2. The rIL-19-treated group took significantly less time and distance to find the platform when compared to the vehicle group (Figures 5(a)–5(c)). There was no statistical difference in the average swimming speed of each group (data not shown). The Probe Trial demonstrated that the vehicle-treated group spent significantly less time in the target quadrant when compared to sham animals, whereas rIL-19-treated animals spent more time in the target quadrant compared to the vehicle group (Figure 5(d)). Thus, rIL-19 improved spatial and reference memory after GMH.

Rotarod and foot fault tests were conducted to evaluate motor coordination on the 28th day after GMH. rIL-19-treated animals demonstrated shorter falling latency on both 5 RMP and 10 RMP accelerating rods compared to the sham group. rIL-19-treatment group had significantly lower falling latencies than the vehicle-treated group (Figure 5(f)). Additionally, rIL-19-treated animals had significantly lower number of foot faults when compared to vehicle (Figure 5(e)). rIL-19 attenuated long-term motor coordination deficits induced by GMH. There was no statistical difference observed between male and female rats (data not shown) during these tests.

### 3.3. rIL-19 Reduced Hematoma Volume and Hemoglobin Content at 72 Hours Post-GMH

**Figure 6:**
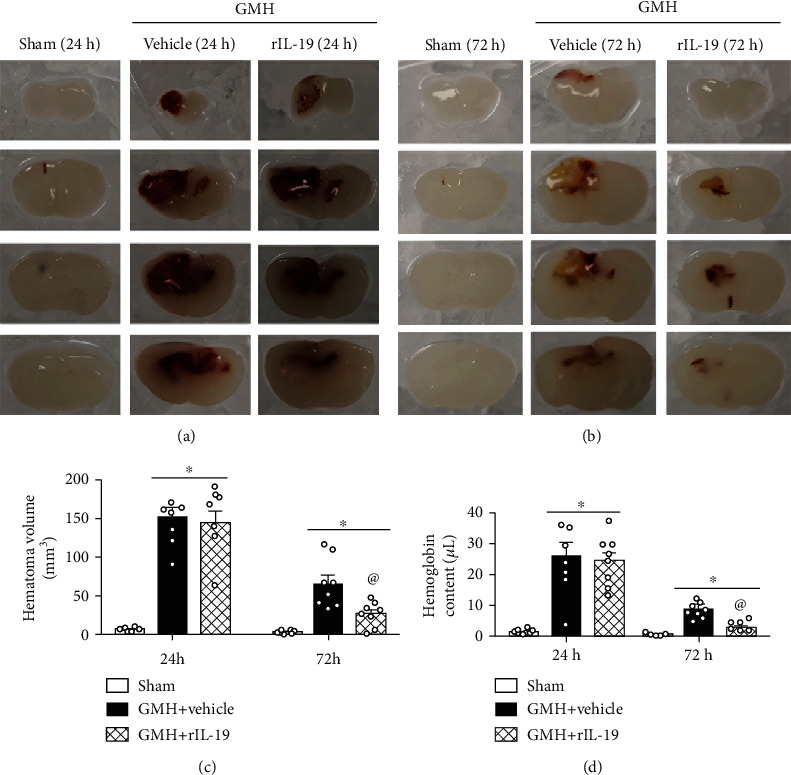
rIL-19 (10 *μ*g/kg, i.n.) treatment reduced hematoma volume and hemoglobin content at 24 hours and 72 hours after GMH. (a) Representative brain sections at 24 hours after GMH. (b) Representative photograph of brain sections at 72 hours after GMH. (c) Hematoma volume. (d) Hemoglobin content. ∗*P* < 0.05 vs. sham, ^@^*P* < 0.05 vs. vehicle, mean ± SEM, one-way ANOVA for (d–f), Tukey's test, *n* = 8/group.

To assess the effect of rIL-19 on hematoma volume, rat pup brains were evaluated at 24 and 72 hours after GMH. At 24 hours, no statistical difference was found between vehicle and rIL-19 treatment groups (Figures 6(a) and 6(c)). At the 72-hour time point, rIL-19 significantly decreased hematoma volume when compared to the vehicle group (Figures 6(b) and 6(c)). The same trend was seen in hemoglobin assay, as rIL-19 treatment significantly decreased hemoglobin content when compared to the vehicle group (Figure 6(d)).

### 3.4. rIL-19 Enhanced the CD163 Expression on Microglia/Macrophages at 72 Hours after GMH

**Figure 7:**
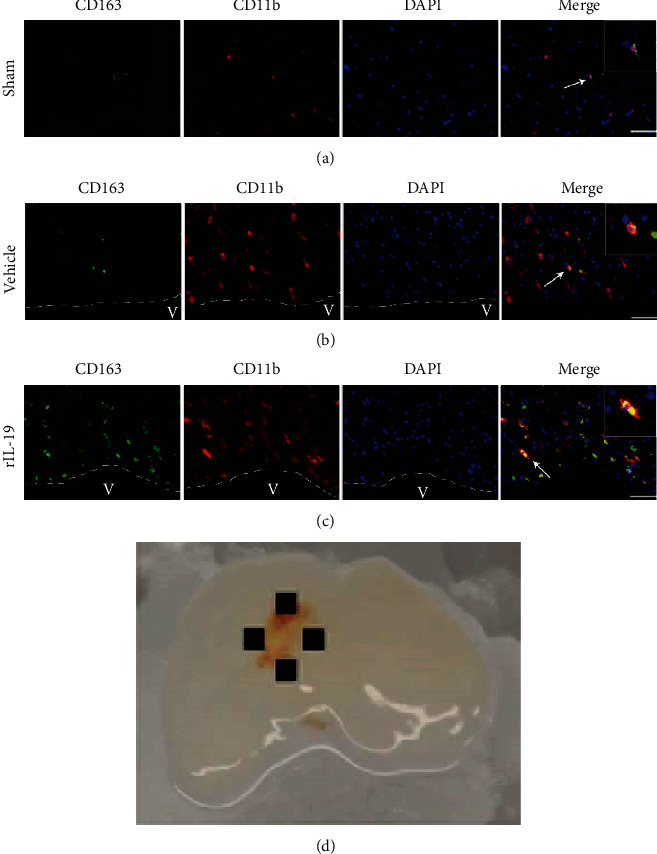
rIL-19 (10 *μ*g/kg, i.n.) treatment increased the number of CD163^+^ cells in microglia/macrophages at 72 hours after GMH. (a) Colocalization of CD163 with CD11b in sham group. (b) Colocalization of CD163 with CD11b in GMH + vehicle group. (c) Colocalization of CD163 with CD11b in GMH + rIL-19 group. (d) Representative GMH brain indicates where IHC images were taken. Scale bar = 50 *μ*m, *n* = 4/group.

Scavenger receptor CD163 was colocalized with CD11b to determine CD163^+^ microglia/macrophages cells around the site of perihematoma and was quantified as previous described in [12]. 72 hours after GMH, the number of microglia/macrophages was significantly increased in the GMH groups when compared to sham (Figure 7). rIL-19-treated group, significantly increased CD163^+^ microglia/macrophages positive cells when compared to sham and vehicle (Figure 7).

### 3.5. rIL-19 Reduced Ventricular Volume and Attenuated Brain Atrophy 28 Days after GMH

**Figure 8:**
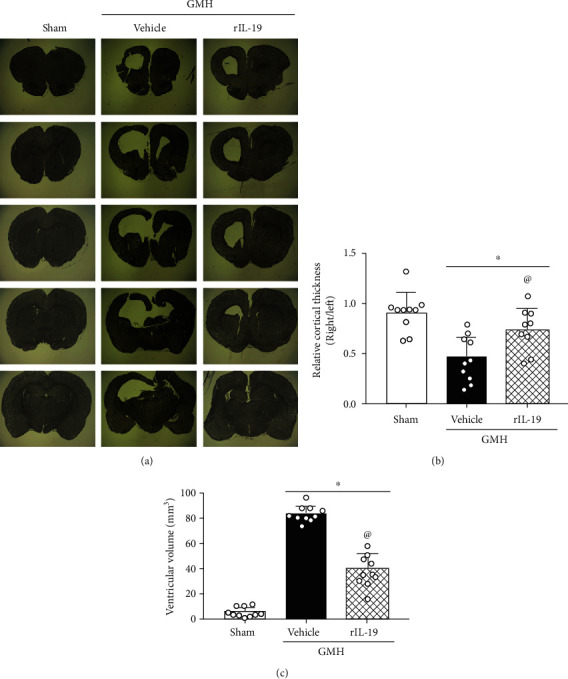
rIL-19 (10 *μ*g/kg, i.n.) treatment reduced the ventricular volume and preserved the cortical thickness of ipsilateral brain 28 days after GMH. (a) Representative Nissl staining images. (b) Ventricular volume. (c) The relative cortical thickness. ∗*P* < 0.05 vs. sham, ^@^*P* < 0.05 vs. vehicle, mean ± SEM, one-way ANOVA, Tukey's test, *n* = 10/group.

Nissl staining was performed to help assess ventricular volume and relative cortical thickness at 28 days after GMH. Compared to sham animals, the vehicle group had significantly increased ventricle dilation and decreased cortical thickness, whereas rIL-19 treatment group significantly reduced ventricular dilation and protected against the loss of cortical thickness (Figure 8).

### 3.6. The Nrf2 Inhibitor ML385 and IL-20R1 CRISPR Inhibited the Signaling Pathway Induced by rIL-19 after GMH

**Figure 9:**
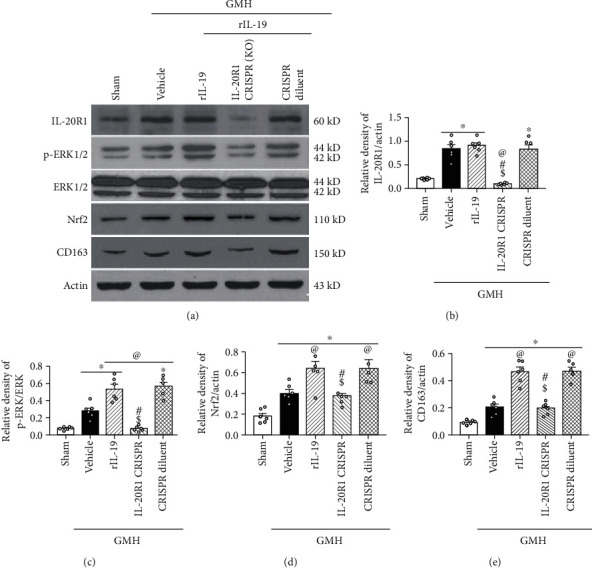
rIL-19 upregulated the proposed signaling pathway while IL-20R1 inhibitor CRISPR (K.O.) reversed the effect of rIL-19 after GMH. (a) The representative bands of IL-20R1, p-ERK, ERK, Nrf2, CD163, and *β*-Actin. (b–e) The quantitative analysis of IL-20R1, p-ERK, Nrf2, and CD163 intervened with IL-20R1 CRISPR-knockdown plasmid. ∗*P* < 0.05 vs. sham, ^@^*P* < 0.05 vs. vehicle, ^#^*P* < 0.05 vs. rIL-19, ^$^*P* < 0.05 vs. rIL-19 + CRISPR diluent. Mean ± SEM, one-way ANOVA, Tukey's test, *n* = 6/group.

**Figure 10:**
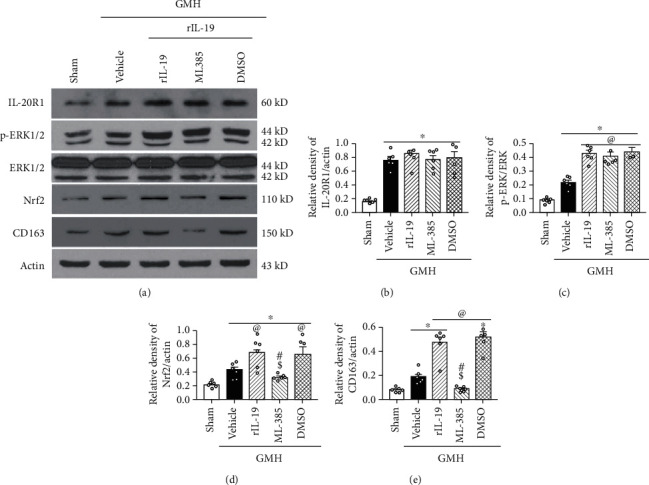
rIL-19 upregulated the expression of p-ERK, Nrf2, and CD163 while the administration of Nrf2 inhibitor ML385 reversed the effect of rIL-19 after GMH. (a) The representative bands of IL-20R1, p-ERK, ERK, Nrf2, CD163, and Actin. (b–e) The quantitative analysis of IL-20R1, p-ERK, Nrf2, and CD163 intervened with ML385. ∗*P* < 0.05 vs. sham, ^@^*P* < 0.05 vs. vehicle, ^#^*P* < 0.05 vs. rIL-19, ^$^*P* < 0.05 vs. rIL-19 + DMSO. Mean ± SEM, one-way ANOVA, Tukey's test, *n* = 6/group.

IL-19 activation of ERK and its downstream protein Nrf2 play a pivotal role in the expression of CD163 and its enhancement of hematoma clearance after GMH. Thus, we evaluated the effects of the rIL-19 on p-ERK, Nrf2, and CD163 at 72 hours postictus. rIL-19 significantly increased p-ERK, Nrf2, and CD163, when compared to sham and vehicle (Figures 9 and 10). Knockdown of IL-20R1 by specific CRISPR plasmid significantly attenuated the expression of IL-20R1, p-ERK, and Nrf2, which was accompanied by a decrease in the expression of CD163 at 72 hours after GMH (Figures 9(a) and 9(b)). Furthermore, the upregulation of Nrf2 by rIL-19 was inhibited by Nrf2 inhibitor ML385, which also demonstrated to significantly reduce the expression of CD163 (Figure 10). Lastly, ML385 had no effects on the expression of upstream proteins IL-19, IL-20R1, and p-ERK.

## 4. Discussion

GMH is a one of the most devastating stroke subtypes for newborns, where the major neurological complications after hemorrhage are neuroinflammation, cerebral palsy, PHH, and motor and cognitive deficits [36]. Currently, GMH research has primarily focused on neuroinflammation [29, 37], reactive astrogliosis [31, 35], blood-brain barrier (BBB) destruction [38, 39], and hematoma resolution [12, 40] which has been closely associated with hematoma. In this study, our focus is on the blood clot which is primary causative factor in GMH-induced secondary brain injury. Here, we are the first to investigate the therapeutic effects of rIL-19 and its role in hematoma clearance by activating IL-20R1 signaling pathway after GMH. The following observations were made: (a) Endogenous protein levels of IL-19 and its receptor IL-20R were significantly expressed postictus, which correlated with an increase in endogenous CD163; (b) IL-20R1 was shown to be expressed on microglia/macrophages cells after GMH; (c) rIL-19 treatment significantly improved short- and long-term neurological function after GMH; (d) rIL-19 treatment significantly reduced GMH-induced ventricular dilation and cortical thickness loss; (e) rIL-19 decreased hematoma volume and hemoglobin content; and (f) IL-20R1 CRISPR-knockdown plasmid and Nrf2 inhibitor ML385 abolished the effects of rIL-19 treatment on the upregulation of the proposed signaling pathway.

Resident microglia and peripheral macrophages express the same set of protein markers, making it difficult to distinguish in CNS [41]. We will refer to resident and recruited microglia/macrophages as one population. Microglia/macrophages are classified into two phenotypes: activated microglia (M1) and alternatively activated microglia (M2) phenotypes. These two phenotypes have different distinct functions after hemorrhagic stroke. Current literature on hematoma clearance has demonstrated that M2 microglia play a pivotal role in this mechanism of action [11, 42]. Three days post-GMH, we demonstrated an increase in the number of CD11b^+^ cells, which is consistent with previous studies [43]. This basic pathological process provides a prerequisite for further research on hematoma clearance in GMH. Scavenger receptor CD163 is primarily expressed on M2 microglia/macrophages and plays an important role in phagocytosis [14, 44]. Increased expression of endogenous CD163 at the 72-hour time point of the time-course study may be the result of the change in the M1/M2 ratio. Hemoglobin (Hb), which is released from metabolized erythrocytes, binds to haptoglobin (Hp) forming a Hb-Hp complex [45]. This complex is recognized by CD163, which results in the engulfment of the hematoma [46, 47]. Thus, targeting CD163 may be beneficial for blood clot clearance after GMH.

IL-19 has been previously reported to play an anti-inflammatory role in various disease models [48–50]. Yet its role in the GMH pathophysiology remains to be elucidated. The IL-19 has been shown to be expressed on various immune cells such as monocytes, B cells, and T cells [51, 52]. Interestingly, IL-19 binds with IL-20 receptor type 1, which specifically contains the IL-20R1 subunit [53]. Like IL-19, we demonstrated that IL-20R type 1 was also expressed on microglia/macrophages after GMH. During the time-course experiments, we saw an inconsistent tendency between IL-20R1 and IL-20R2 after GMH, which may be related to the IL-20R type 2. It is reported that IL-19 has a higher affinity to IL-20R type 1, since IL-19 showed higher affinity to bind IL-20R2 subunit than other ligands such as IL-20 [54].

Following the binding of IL-19 with IL-20R1 and IL-20R2, the cytoplasmic tail of IL-20R1 directly initiated the phosphorylation of ERK1/2 [18] or indirectly stimulated the phosphorylation of the molecule through the JAK/STAT signaling pathway [55, 56]. Mounting evidence suggest that phosphorylated ERK may stimulate the expression of Nrf2, thereby blocking the Nrf2-binding domain with Keap-1 [57, 58]. Nrf2 then enters into the nucleus to increase the transcription activity of CD163 [59, 60]. Experiment 4 demonstrated the synchronization IL20R1/ERK/Nrf2/CD163 signaling pathway in sham, vehicle, and rIL-19 treatment groups. To study the rIL-19 mechanism of action on hematoma clearance, we evaluated its effects on the IL-20R1/ERK/Nrf2 signaling axis with the use of inhibitors. IL-20R1 CRISPR-knockdown plasmid ameliorated the therapeutic effects of rIL-19 on hematoma clearance, which effected the expression of CD163 and Nrf2. Nrf2 inhibitor ML385 also reversed the effects of rIL-19 on the expression of CD163. This study demonstrated that rIL-19 promoted the hematoma clearance after GMH through the activation of IL-20R1/ERK/Nfr2 signaling pathway.

Although we present compelling evidence, there are some limitations to this study. First, we only tested the expression of proteins and did not measure the gene activity of each target molecule. Second, we did not measure the concentration of rIL-19 in serum or brain tissue. Third, it is possible that there are other proteins involved in hematoma clearance, and the present experimental result may be the part of a larger pathway. Lastly, rIL-19 and ERK have been shown to have anti-inflammatory and proregeneration effects in other models [61]. Because of this, rIL-19 promotion of hematoma clearance cannot be entirely attributed as the sole cause of neuroprotection after GMH.

## 5. Conclusion

In this current study, we demonstrated that rIL-19 treatment attenuated neurological deficits and promoted hematoma clearance after GMH in neonatal rat pups. The protective effects were mediated through the activation of IL-20R1/ERK/Nrf2 signaling pathway. Our study is the first to demonstrate rIL-19 effects on hematoma clearance, providing new insight for noninvasive therapeutic strategies for the management of GMH.

## Data Availability

The data support the findings of this study and are available from the corresponding author upon reasonable request.

## Abbreviations

BBB: Blood-brain barrier
CRISPR: Clustered regularly interspaced short palindromic repeats
CSF: Cerebrospinal fluid
DAPI: 4',6-diamidino-2-phenylindole
DMSO: Dimethyl sulfoxide
ERK: Extracellular regulated protein kinases
GMH: Germinal matrix hemorrhage
IF: Immunofluorescence
IL-19: Interleukin-19
IL-20R: Interleukin-20 receptor
Nrf2: Nuclear factor erythroid-2-related factor 2
p-ERK: Phosphorylated extracellular regulated protein kinases
PHH: Posthemorrhagic hydrocephalus
PRX-2: Peroxiredoxin 2
RBS: Red blood cell
rIL-19: Recombination Interleukin-19
WB: Western blot.

## References

[1] Tortora D., Severino M., Malova M. (2017). Differences in subependymal vein anatomy may predispose preterm infants to GMH-IVH. *Archives of Disease in Childhood-Fetal and Neonatal Edition*.

[2] Ramenghi L. A., Fumagalli M., Groppo M. (2011). Germinal matrix hemorrhage: intraventricular hemorrhage in very-low-birth-weight infants: the independent role of inherited thrombophilia. *Stroke*.

[3] Flores J. J., Klebe D., Tang J., Zhang J. H. (2020). A comprehensive review of therapeutic targets that induce microglia/macrophage-mediated hematoma resolution after germinal matrix hemorrhage. *Journal of Neuroscience Research*.

[4] Ballabh P. (2014). Pathogenesis and prevention of intraventricular hemorrhage. *Clinics in Perinatology*.

[5] Ballabh P. (2010). Intraventricular hemorrhage in premature infants: mechanism of disease. *Pediatric Research*.

[6] Cao S., Zheng M., Hua Y., Chen G., Keep R. F., Xi G. (2016). Hematoma changes during clot resolution after experimental intracerebral hemorrhage. *Stroke*.

[7] Zhao X., Song S., Sun G. (2009). Neuroprotective role of haptoglobin after intracerebral hemorrhage. *Journal of Neuroscience*.

[8] Bian L., Zhang J., Wang M., Keep R. F., Xi G., Hua Y. (2020). Intracerebral hemorrhage-induced brain injury in rats: the role of extracellular peroxiredoxin 2. *Translational Stroke Research*.

[9] Wang G., Li T., Duan S.-n., Dong L., Sun X.-g., Xue F. (2018). PPAR-*γ* promotes hematoma clearance through haptoglobin-hemoglobin-CD163 in a rat model of intracerebral hemorrhage. *Behavioural Neurology*.

[10] Bosche B., Mergenthaler P., Doeppner T. R., Hescheler J., Molcanyi M. (2020). Complex clearance mechanisms after intraventricular hemorrhage and rt-PA treatment: a review on clinical trials. *Translation Stroke Research*.

[11] Lan X., Han X., Li Q., Yang Q. W., Wang J. (2017). Modulators of microglial activation and polarization after intracerebral haemorrhage. *Nature Reviews Neurology*.

[12] Flores J. J., Klebe D., Rolland W. B., Lekic T., Krafft P. R., Zhang J. H. (2016). PPAR*γ*-induced upregulation of CD36 enhances hematoma resolution and attenuates long-term neurological deficits after germinal matrix hemorrhage in neonatal rats. *Neurobiology of Disease*.

[13] Imam M., Zhang S., Ma J., Wang H., Wang F. (2017). Antioxidants mediate both iron homeostasis and oxidative stress. *Nutrients*.

[14] Garton T., Keep R. F., Hua Y., Xi G. (2017). CD163, a hemoglobin/haptoglobin scavenger receptor, after intracerebral hemorrhage: functions in microglia/macrophages versus neurons. *Translational Stroke Research*.

[15] Ray M., Gabunia K., Vrakas C. N. (2018). Genetic deletion of IL-19 (interleukin-19) exacerbates atherogenesis inIl19−/−×Ldlr−/−Double knockout mice by dysregulation of mRNA stability protein HuR (human antigen R). *Arteriosclerosis Thrombosis and Vascular Biology*.

[16] Ellison S., Gabunia K., Kelemen S. E. (2013). Attenuation of experimental atherosclerosis by interleukin-19. *Arteriosclerosis Thrombosis and Vascular Biology*.

[17] Xie W., Fang L., Gan S., Xuan H. (2016). Interleukin-19 alleviates brain injury by anti-inflammatory effects in a mice model of focal cerebral ischemia. *Brain Research*.

[18] Burmeister A. R., Marriott I. (2018). The interleukin-10 family of cytokines and their role in the CNS. *Frontiers in Cellular Neuroscience*.

[19] Moniruzzaman M., Wang R., Jeet V., McGuckin M. A., Hasnain S. Z. (2019). Interleukin (IL)-22 from IL-20 subfamily of cytokines induces colonic epithelial cell proliferation predominantly through ERK1/2 pathway. *International Journal of Molecular Sciences*.

[20] Pilipović I., Stojić-Vukanić Z., Prijić I., Jasnić N., Leposavić G. (2020). Propranolol diminished severity of rat EAE by enhancing immunoregulatory/protective properties of spinal cord microglia. *Neurobiology of Disease*.

[21] Landis R. C., Quimby K. R., Greenidge A. R. (2018). M1/M2 macrophages in diabetic nephropathy: Nrf2/HO-1 as therapeutic targets. *Current Pharmaceutical Design*.

[22] Li P., Zhao G., Ding Y. (2019). Rh-IFN-*α* attenuates neuroinflammation and improves neurological function by inhibiting NF-*κ*B through JAK1-STAT1/TRAF3 pathway in an experimental GMH rat model. *Brain Behavior and Immunity*.

[23] Gabunia K., Ellison S., Kelemen S. (2016). IL-19 halts progression of atherosclerotic plaque, polarizes, and increases cholesterol uptake and efflux in macrophages. *American Journal of Pathology*.

[24] Richards J., Gabunia K., Kelemen S. E., Kako F., Choi E. T., Autieri M. V. (2015). Interleukin-19 increases angiogenesis in ischemic hind limbs by direct effects on both endothelial cells and macrophage polarization. *Journal of Molecular and Cellular Cardiology*.

[25] Xian P., Hei Y., Wang R. (2019). Mesenchymal stem cell-derived exosomes as a nanotherapeutic agent for amelioration of inflammation-induced astrocyte alterations in mice. *Theranostics*.

[26] Ohtomo R., Kinoshita K., Ohtomo G. (2020). Treadmill exercise suppresses cognitive decline and increases white matter oligodendrocyte precursor cells in a mouse model of prolonged cerebral hypoperfusion. *Translational Stroke Research*.

[27] Jackson L., Dong G., Althomali W. (2020). Delayed administration of angiotensin II type 2 receptor (AT2R) agonist compound 21 prevents the development of post-stroke cognitive impairment in diabetes through the modulation of microglia polarization. *Translational Stroke Research*.

[28] Tanaka M., Ogaeri T., Samsonov M., Sokabe M. (2019). The 5*α*-reductase inhibitor finasteride exerts neuroprotection against ischemic brain injury in aged male rats. *Translational Stroke Research*.

[29] Zhang Y., Xu N., Ding Y. (2018). Chemerin suppresses neuroinflammation and improves neurological recovery via CaMKK2/AMPK/Nrf2 pathway after germinal matrix hemorrhage in neonatal rats. *Brain Behavior, and Immunity*.

[30] Lekic T., Manaenko A., Rolland W. (2012). Rodent neonatal germinal matrix hemorrhage mimics the human brain injury, neurological consequences, and post-hemorrhagic hydrocephalus. *Experimental Neurology*.

[31] Liu S.‐. P., Huang L., Flores J. (2019). Secukinumab attenuates reactive astrogliosis via IL-17RA/(C/EBP*β*)/SIRT1 pathway in a rat model of germinal matrix hemorrhage. *CNS Neuroscience & Therapeutics*.

[32] Wan W., Ding Y., Xie Z. (2019). PDGFR-*β* modulates vascular smooth muscle cell phenotype via IRF-9/SIRT-1/NF-*κ*B pathway in subarachnoid hemorrhage rats. *Journal of Cerebral Blood Flow and Metabolism*.

[33] Klebe D., Flores J. J., McBride D. W. (2017). Dabigatran ameliorates post-haemorrhagic hydrocephalus development after germinal matrix haemorrhage in neonatal rat pups. *Journal of Cerebral Blood Flow and Metabolism*.

[34] Li Q., Ding Y., Krafft P. (2018). Targeting germinal matrix hemorrhage-induced overexpression of sodium-coupled bicarbonate exchanger reduces posthemorrhagic hydrocephalus formation in neonatal rats. *Journal of the American Heart Association*.

[35] Ding Y., Zhang T., Wu G. (2019). Astrogliosis inhibition attenuates hydrocephalus by increasing cerebrospinal fluid reabsorption through the glymphatic system after germinal matrix hemorrhage. *Experimental Neurology*.

[36] Zhang S., Wang X., Li S. (2020). Outcome of two pairs of monozygotic twins with pleuropulmonary blastoma: case report. *Italian Journal of Pediatrics*.

[37] Tao Y., Li L., Jiang B. (2016). Cannabinoid receptor-2 stimulation suppresses neuroinflammation by regulating microglial M1/M2 polarization through the cAMP/PKA pathway in an experimental GMH rat model. *Brain Behavior and Immunity*.

[38] Dave J. M., Mirabella T., Weatherbee S. D., Greif D. M. (2018). Pericyte ALK5/TIMP3 axis contributes to endothelial morphogenesis in the developing brain. *Developmental Cell*.

[39] Li L., Tao Y., Tang J. (2015). A cannabinoid receptor 2 agonist prevents thrombin-induced blood-brain barrier damage via the inhibition of microglial activation and matrix metalloproteinase expression in rats. *Translational Stroke Research*.

[40] Zhang Y., Ding Y., Lu T. (2018). Bliverdin reductase-A improves neurological function in a germinal matrix hemorrhage rat model. *Neurobiology of Disease*.

[41] Carson M. J., Bilousova T. V., Puntambekar S. S., Melchior B., Doose J. M., Ethell I. M. (2007). A rose by any other name? The potential consequences of microglial heterogeneity during CNS health and disease. *Neurotherapeutics*.

[42] Ni W., Mao S., Xi G., Keep R. F., Hua Y. (2016). Role of erythrocyte CD47 in intracerebral hematoma clearance. *Stroke*.

[43] Chang C. F., Wan J., Li Q., Renfroe S. C., Heller N. M., Wang J. (2017). Alternative activation-skewed microglia/macrophages promote hematoma resolution in experimental intracerebral hemorrhage. *Neurobiology of Disease*.

[44] Newell E., Shellington D. K., Simon D. W. (2015). Cerebrospinal fluid markers of macrophage and lymphocyte activation after traumatic brain injury in children. *Pediatric Critical Care Medicine*.

[45] Andersen C. B. F., Stødkilde K., Sæderup K. L. (2017). Haptoglobin. *Antioxidants & Redox Signaling*.

[46] Thomsen J. H., Etzerodt A., Svendsen P., Moestrup S. K. (2013). The haptoglobin-CD163-heme oxygenase-1 pathway for hemoglobin scavenging. *Oxidative Medicine and Cellular Longevity*.

[47] Savman K., Nilsson U. A., Blennow M., Kjellmer I., Whitelaw A. (2001). Non-protein-bound iron is elevated in cerebrospinal fluid from preterm infants with posthemorrhagic ventricular dilatation. *Pediatric Research*.

[48] Rong B., Liu Y., Li M., Fu T., Gao W., Liu H. (2018). Correlation of serum levels of HIF-1*α* and IL-19 with the disease progression of COPD: a retrospective study. *International Journal of Chronic Obstructive Pulmonary Disease*.

[49] Fonseca-Camarillo G., Furuzawa-Carballeda J., Granados J., Yamamoto-Furusho J. K. (2014). Expression of interleukin (IL)-19 and IL-24 in inflammatory bowel disease patients: a cross-sectional study. *Clinical and Experimental Immunology*.

[50] Azuma Y. T., Nakajima H., Takeuchi T. (2011). IL-19 as a potential therapeutic in autoimmune and inflammatory diseases. *Current Pharmaceutical Design*.

[51] Gallagher G., Dickensheets H., Eskdale J. (2000). Cloning, expression and initial characterisation of interleukin-19 (IL-19), a novel homologue of human interleukin-10 (IL-10). *Genes and Immunity*.

[52] Wolk K., Kunz S., Asadullah K., Sabat R. (2002). cutting edge: immune cells as sources and targets of the IL-10 family members?. *Journal of Immunology*.

[53] Chen J., Caspi R. R., Chong W. P. (2018). IL-20 receptor cytokines in autoimmune diseases. *Journal of Leukocyte Biology*.

[54] Logsdon N. J., Deshpande A., Harris B. D., Rajashankar K. R., Walter M. R. (2012). Structural basis for receptor sharing and activation by interleukin-20 receptor-2 (IL-20R2) binding cytokines. *Proceedings of the National Academy of Sciences of the United States of America*.

[55] Hsu Y. H., Wu C. Y., Hsing C. H., Lai W. T., Wu L. W., Chang M. S. (2015). Anti-IL-20 monoclonal antibody suppresses prostate cancer growth and bone osteolysis in murine models. *PLoS One*.

[56] Lee S. J., Cho S. C., Lee E. J. (2013). Interleukin-20 promotes migration of bladder cancer cells through extracellular signal-regulated kinase (ERK)-mediated MMP-9 protein expression leading to nuclear factor (NF-*κ*B) activation by inducing the up-regulation of p21(WAF1) protein expression. *Journal of Biological Chemistry*.

[57] Khan N. M., Haseeb A., Ansari M. Y., Devarapalli P., Haynie S., Haqqi T. M. (2017). Wogonin, a plant derived small molecule, exerts potent anti-inflammatory and chondroprotective effects through the activation of ROS/ERK/Nrf2 signaling pathways in human osteoarthritis chondrocytes. *Free Radical Biology & Medicine*.

[58] Li X., He P., Wang X. L. (2018). Sulfiredoxin-1 enhances cardiac progenitor cell survival against oxidative stress via the upregulation of the ERK/NRF2 signal pathway. *Free Radical Biology & Medicine*.

[59] Boyle J. J., Johns M., Lo J. (2011). Heme induces heme oxygenase 1 via Nrf2. *Arteriosclerosis Thrombosis and Vascular Biology*.

[60] Wang G., Wang L., Sun X.-g., Tang J. (2017). Haematoma scavenging in intracerebral haemorrhage: from mechanisms to the clinic. *Journal of Cellular and Molecular Medicine*.

[61] Liu L., Yuan H., Yi Y. (2018). Ras-related C3 botulinum toxin substrate 1 promotes axonal regeneration after stroke in mice. *Translational Stroke Research*.

